# Impact of rigid cardiac motion on the accuracy of electrocardiographic imaging

**DOI:** 10.3389/fphys.2025.1560527

**Published:** 2025-05-15

**Authors:** Xiafeng Zhang, Kaiyu Chen, Yucheng Wang, Wei Li, Tingcun Wei, Shaoxi Wang

**Affiliations:** School of Microelectronics, Northwestern Polytechnical University, Xi’an, China

**Keywords:** electrocardiographic imaging (ECGI), cardiac rigid motion, translation, rotation, inverse solution

## Abstract

**Introduction:**

Electrocardiographic Imaging (ECGI) offers a non-invasive approach to reconstruct cardiac electrical activity. However, the inverse problem of ECGI is highly ill-conditioned, making it sensitive to errors. In practice, rigid displacements of the heart during beating introduce geometric errors into the ECGI problem. This study aims to investigate the impact of cardiac rigid motion on the accuracy of ECGI.

**Methods:**

We employed the Boundary Element Method (BEM) to solve the forward problem and the Tikhonov method to address the inverse problem. We utilized a dataset from the CRVTI/SCI Institute, which involves Langendorff-perfused dog hearts suspended in a torso-shaped tank. Based on clinical experience, six different types of cardiac movement patterns, including translations and rotations, were designed to assess the impact of various displacements on the accuracy of the ECGI solution.

**Results:**

Our study found that among the translational and rotational movements of the heart, rotational motion should be prioritized for attention, as it caused significantly stronger changes in ECGI correlation coefficient (CC) and relative error (RE) than translational motion. Among the translations along the coordinate axes, movement along the y-axis (anterior-posterior movement within the chest cavity) had the least impact. For rotational movements, rolling had the least impact, yaw had moderate impact, and pitch had the greatest impact.

**Conclusion:**

The inverse solution of ECGI demonstrates a certain robustness to changes in heart position, with CC changes of less than 2% for 10 mm displacements and less than 5% for 10° rotations. This suggests that ECGI changes due to cardiac geometric motion can be disregarded within a certain range.

## 1 Introduction

Electrocardiographic Imaging (ECGI) is a promising medical technology that employs body surface electrocardiograms (ECGs) and a torso volume conductor model to non-invasively reconstruct cardiac electrical activity (Cluitmans et al., 2018). This technology allows for the identification of extracellular potentials on the endocardium and epicardium (Kalinin et al., 2019; Pedron-Torrecilla et al., 2016), activation time sequences on the cardiac surface (Van der Waal et al., 2021; van der Waal et al., 2020; Zhou et al., 2019), and transmembrane voltages of the heart (Zaman et al., 2021; Schuler et al., 2018), among other details. ECGI has been clinically and experimentally applied to a range of pathologies and applications, including locating sites of premature activation, identifying arrhythmic circuits, preoperative planning, and guiding ablation procedures (Cluitmans et al., 2018; Salinet et al., 2021). Contemporary ECGI research aims to address novel diseases and improve accuracy, stability, and practicality (Dogrusoz et al., 2019; Schuler et al., 2022). However, one source of input error in ECGI systems, namely errors in modeling heart position, has not been effectively addressed in limited studies, and its impact on the accuracy of ECGI remains unclear (Rodrigo et al., 2018; Coll-Font and Brooks, 2018; Bergquist et al., 2022; Bergquist et al., 2023).

**TABLE 1:** Summary of experimental results.

Motion type	Displacement/ Rotation	sCC change (%)	sRE change (%)	tCC change (%)	tRE change (%)	PT change (ms)
Translation (X-axis: Lateral)	−30 mm	−4.89% ± 2.85%	2.07% ± 1.67%	−2.68% ± 2.16%	3.56% ± 2.44%	−1.53 ± 0.75 ms
	−15 mm	−1.60% ± 0.86%	0.60% ± 0.53%	−0.61% ± 0.6%	1.07% ± 0.74%	−0.73 ± 0.55 ms
	+15 mm	0.65% ± 0.83%	0.15% ± 0.31%	−0.56% ± 0.56%	0.54% ± 0.61%	0.74 ± 0.53 ms
	+30 mm	0.42% ± 2.11%	0.92% ± 0.94%	−1.73% ± 1.30%	2.35% ± 2.03%	1.23 ± 0.51 ms
Translation (Y-axis: Anterior-Posterior)	−30 mm	0.20% ± 1.47%	0.62% ± 0.66%	−0.89% ± 0.42%	1.97% ± 1.49%	−0.12 ± 0.64 ms
	−15 mm	0.78% ± 0.43%	−0.10% ± 0.14%	0.12% ± 0.36%	0.48% ± 0.46%	−0.08 ± 0.32 ms
	+15 mm	−1.25% ± 0.96%	0.67% ± 0.51%	−0.40% ± 0.49%	0.64% ± 0.41%	0.15 ± 0.35 ms
	+30 mm	−2.57% ± 2.23%	1.59% ± 1.18%	−0.87% ± 0.83%	2.00% ± 1.07%	0.44 ± 0.55 ms
Translation (Z-axis: Cranio-Caudal)	−30 mm	−4.66% ± 3.47%	2.46% ± 1.65%	−5.26% ± 1.40%	6.94% ± 2.51%	−1.47 ± 0.59 ms
	−15 mm	−1.08% ± 1.15%	0.63% ± 0.49%	−1.27% ± 0.18%	2.25% ± 0.66%	−0.94 ± 0.46 ms
	+15 mm	−1.56% ± 0.74%	0.74% ± 0.46%	−0.18% ± 0.46%	0.42% ± 0.83%	1.01 ± 0.30 ms
	+30 mm	−5.31% ± 2.72%	2.52% ± 1.55%	−1.80% ± 1.26%	2.91% ± 2.72%	2.24 ± 0.48 ms
Rotation (Pitch: Forward-Backward Tilt)	−30°	−14.48% ± 7.93%	6.44% ± 4.61%	−11.34% ± 2.40%	12.40% ± 6.66%	1.29 ± 0.67 ms
	−15°	−4.05% ± 2.63%	1.94% ± 1.52%	−3.88% ± 0.89%	3.99% ± 2.59%	0.54 ± 0.55 ms
	+15°	−5.75% ± 3.10%	2.49% ± 1.84%	−3.19% ± 0.52%	3.83% ± 1.80%	−0.21 ± 0.65 ms
	+30°	−19.57% ± 10.14%	7.90% ± 5.59%	−17.00% ± 3.37%	13.45% ± 6.33%	0.25 ± 0.99 ms
Rotation (Yaw: Left-Right Rotation)	−30°	−12.78% ± 6.32%	4.90% ± 3.40%	−12.52% ± 2.65%	6.87% ± 3.08%	0.49 ± 0.69 ms
	−15°	−5.11% ± 3.05%	2.04% ± 1.56%	−4.00% ± 1.70%	1.71% ± 0.88%	0.06 ± 0.57 ms
	+15°	1.01% ± 1.72%	−0.45% ± 0.68%	−1.82% ± 0.66%	2.18% ± 1.57%	−0.29 ± 0.54 ms
	+30°	−3.63% ± 2.55%	1.62% ± 1.05%	−6.40% ± 0.78%	6.71% ± 3.95%	−0.55 ± 0.47 ms
Rotation (Roll: Torsion around Septal Axis)	−30°	−6.30% ± 4.46%	2.56% ± 2.13%	−1.10% ± 1.66%	−0.67% ± 1.20%	0.09 ± 0.42 ms
	−15°	−1.34% ± 1.79%	0.62% ± 0.74%	1.59% ± 0.59%	−1.77% ± 1.11%	0.19 ± 0.32 ms
	+15°	−3.12% ± 0.82%	1.07% ± 0.55%	−3.88% ± 0.47%	3.60% ± 1.77%	−0.13 ± 0.51 ms
	+30°	−9.39% ± 2.11%	3.17% ± 1.58%	−7.95% ± 1.42%	8.52% ± 3.93%	−0.26 ± 0.51 ms

The implementation of ECGI can be divided into two steps: the forward model and the inverse problem. The forward model describes the distribution of body surface potential (BSP) mapping given a particular representation of the cardiac bioelectric source, whose input is a mathematical model of the electrical activity of the heart and the geometry, conductivity, and relative position of the organs in the trunk. The inverse problem estimates the parameters of the source model given a specific set of BSP signals, with the forward model serving as an input for the inverse problem. The ECGI inverse problem is an ill-posed estimation problem, meaning that the solution is highly sensitive to small fluctuations or noise in the input or small errors in the model (Figuera et al., 2016).

A frequent source of poorly controlled input error for ECGI formulations is inaccuracy in the geometric model used in the forward problem (Bergquist et al., 2023). An accurate three-dimensional model of the torso and heart requires the use of medical imaging techniques, such as MRI or CT, whose images must be manually or semi-automatically segmented. MRI or CT images are typically captured at a single phase of the respiratory and cardiac cycles, often before or after electrocardiogram acquisition. Changes in respiration, heartbeat, and body position inevitably introduce uncertainties in the heart’s position, manifesting as noise within the imaging modalities. Consequently, the geometric models derived from these images cannot account for variations in heart position (Bergquist et al., 2022).

Studies have shown that ECGI is sensitive to geometric model errors. Several studies have examined how errors in geometric models, particularly those stemming from uncertainties in heart position, can adversely affect the forward solution. For example, Swenson et al. and Bear et al. explored how such errors influence the accuracy of the forward model (Swenson et al., 2011; Bear et al., 2015). Cluitmans and Volders specifically investigated how torso geometry accuracy impacts the noninvasive reconstruction of electrical activation and recovery in the inverse problem (Cluitmans and Volders, 2017). More recently, Bergquist et al. focused on quantifying the uncertainty of cardiac position variability (Bergquist et al., 2023), while Molero et al. evaluated the robustness of inverse solutions in the face of atrial morphology and location uncertainty (Molero et al., 2023). Similarly, Corrado et al. examined the impact of atrial shape uncertainty on arrhythmia prediction (Corrado et al., 2023). In addition, several studies have sought to improve ECGI reconstruction quality by correcting heart position and minimizing geometric input errors in the forward model. Notable works in this area include those by Gisbert et al. (2020), Bergquist et al. (2022). While these studies have contributed significantly to enhancing model accuracy, fewer studies have addressed the effects of persistent cardiac rigid motion on the inverse problem.

In this paper we evaluated the impact of cardiac motion on the accuracy of the inverse problem in ECGI. Specifically, we analyzed the effect of modeling errors arising from changes in the geometric position of the heart (translations and rotations across six dimensions) on ECGI. Utilizing the dataset comprising Langendorff-perfused dog hearts, we have quantified the accuracy of the ECGI inverse solution under conditions of uncertainty in heart position, focusing on aspects such as temporal and spatial correlation, as well as variations in electrogram morphology’s peak values and peak times.

## 2 Methods

### 2.1 Forward and inverse problems

We assume that the volume between the epicardial surface ( $S_{H}$ ) and the body surface ( $S_{B}$ ) is a source-free homogeneous volume conductor, then the forward problem can be regarded as a quasi-static problem. The relationship between electric potentials on the boundary $S_{H}$ and $S_{B}$ can be represented by

$$\Phi_{B}=A_{BH}\Phi_{H}$$ (1)

where $\Phi_{B}$ and $\Phi_{H}$ represents the potential on $S_{H}$ and $S_{B}$ respectively, and $A_{BH}$ is the transfer matrix, which is computed using the boundary element method (BEM) for a homogeneous torso, as detailed in (Barr et al. (1977), Horacek and Clements (1997).

In the forward problem, the epicardial potential distributions electrograms ( $\Phi_{H}$ ) is given, the BSPs ( $\Phi_{B}$ ) can be calculated directly by Equation 1. In the inverse problem, given the potential $\Phi_{B}$ of the torso surface, it is very complicated to solve $\Phi_{H}$ directly by the linear model. The matrix $A_{BH}$ may not be a square matrix, and it's always highly ill-conditioned. Therefore the zero-order Tikhonov regularization method, which is one of the most widely used techniques for solving inverse problems (Figuera et al., 2016), is used to stabilize the solution by punishing its complexity. The solution can be expressed as Equation 2:

$${\hat{\Phi }}_{H}=\underset{\Phi_{H}}{\mathrm{argmin}}\left\{{\left\|A_{BH}\Phi_{H}-\Phi_{B}\right\|}_{2}^{2}|+|\lambda^{2}|{\left\|\Phi_{H}\right\|}_{2}^{2}\right\}$$ (2)

where λ is the regularization parameter and is determined by the L-curve method (Rodrigo et al., 2018). The estimated value of the inverse problem is:

$${\hat{\Phi }}_{H}={\left(A_{BH}^{T}A_{BH}+\lambda^{2}I\right)}^{-1}A_{BH}^{T}\Phi_{B}$$ (3)

where I is the identity matrix. Based on Equation 3, we can estimate the epicardial potential distribution $\Phi_{H}$ in the inverse problem.

### 2.2 Experimental data sets

The study utilized data recorded in a torso-tank canine experiment conducted at the Cardiovascular Research and Training Institute (CVRTI), University of Utah. The experiment was based on an improved Langendorff perfusion system in which isolated canine hearts were wrapped in a rigid pericardial electrode cage and suspended in a human-shaped torso tank (Bergquist et al., 2021b). The recordings included epicardial electrograms (EGMs) from a 256-electrode pericardial electrode cage and BSPs from 192 electrodes embedded in the tank surface. The experimental data are published on EDGAR, an open source database of the Consortium for ECG Imaging (CEI) (Aras et al., 2015). We extended the experimental data set using the heart and body surface geometry and the epicardial electrograms recorded from the above experiments, as described below. Initially, a series of geometric models representing the heart-torso were developed through adjustments to the position and shape of the pericardial electrode cage. Subsequently, combined with the homogeneous torso model, the forward model is calculated using the BEM. Finally, we synthesized corresponding BSPs utilizing both the forward model and epicardial EGMs recorded by the pericardial electrode cage. Notably, Gaussian white noise with a SNR of 10 dB was incorporated into the synthesized BSP signals prior to any filtering processes. Ultimately, we generated a synthetic dataset comprising 163 data sets. This synthetic dataset exclusively contains sinus rhythm signals, upon which subsequent experiments will be conducted.

### 2.3 Description of the experiments

**FIGURE 1:**
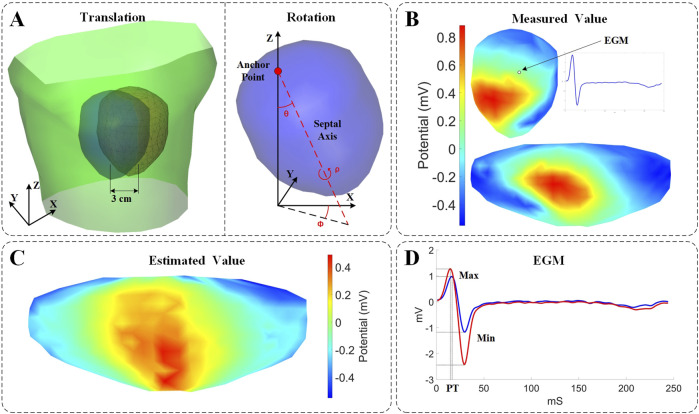
Experimental setup and evaluation description. **(A)** Parametrization describing the motion coordinates. Left: Schematic illustration of the torso surface (green), the pericardial cage in its original position (blue), and the pericardial cage in its displaced position (red). Right: Rotational angles defined on the heart. Pitch (θ): the angle formed between the Z axis and the septal axis. Yaw (φ): the angle formed between the septal axis in the X/Y plane and the X-coordinate. Roll (ρ): the rotation of the heart around the septal axis. **(B)** 3D and 2D displays of potential measurements on the surface of the heart. **(C)** Estimated values from ECGI inverse calculation (time instant = 8). **(D)** Comparison of measured values (blue) and estimated values (red) of the EGM.

To evaluate the impact of cardiac rigid motion on the accuracy of the ECGI inverse problem, we used the heart’s inherent periodic beating as a basis to decompose cardiac motion into translational and rotational motions. Then, we conducted experiments separately for each type of motion. We calculated EGMs at various cardiac positions using BSP signals. Subsequently, we evaluated the changes in correlation coefficients, maximum values, minimum values, and peak times of the EGMs reconstructed from different cardiac positions, as illustrated in Figure 1. The study on the impact of different cardiac motions on the accuracy of the ECGI inverse problem is presented as follows:

Translation Motion: A single displacement along the X, Y, and Z axes. Due to anatomical model constraints, the displacement range is limited from −30 to 30 mm, with a step size of 5 mm. It was evaluated in a subset of 163 models (with 163 distinct models for each axis) by assessing the correlation coefficients, extreme values, and peak times of the EGMs, which were obtained through inverse calculations with gradual displacement along each axis.

Rotational Motion: To effectively describe the rotation of the real heart, the heart’s rotation is defined based on two anatomical references: as illustrated in Figure 1, one is an anchor point placed at the suspended position of the heart, and the other is a septal axis, which extends from the anchor point through the septum of the heart to the apex (Coll-Font and Brooks, 2018). Based on these references, the rotational angle is defined as follows:

• Pitch (θ): the angle formed between the Z axis and the septal axis.
• Yaw (φ): the angle formed between the septal axis projected on the X/Y plane and the X axis.
• Roll (ρ): the rotation of the heart around the septal axis.

The evaluation was conducted across all models, wherein the three parameters underwent gradual rotation in increments of 5° from −30° to 30°. During yaw rotation, θ is set to 15°, and during roll rotation, θ is set to 15° with φ also set to 15°. This configuration allows the pericardial cage structure to be suspended at an angle within the torso tank, more accurately mimicking the actual relative position of the heart and torso. Additionally, it prevents yaw and roll rotations from inadvertently resulting in a rotation primarily around the Z-axis, which would undermine the experiment’s validity.

### 2.4 Evaluation metrics

The Pearson’s correlation coefficient (CC) and relative error (RE) were used to evaluate the impact of cardiac rigid motion on ECGI, by comparing the real measured data with the analytical results of the inverse solutions after accounting for cardiac exercise. The CC reflects the degree of spatial discrepancy between the estimated and measured quantities, while the RE indicates the percentage deviation of the estimates. These two metrics can be employed in two versions: (i) Temporal version: For each node, the CC (or RE) is computed across all time instances, followed by calculating the average CC (or RE) among the nodes; (ii) Spatial version: For each time instant, the CC (or RE) is computed using all nodes, and subsequently, the average CC (or RE) across time instances is determined. Finally, statistics for CC (or RE) are conducted across all models. Temporal CC or RE (tCC or tRE) assesses the reconstruction of EGMs morphology within each lead, while spatial CC or RE (sCC or sRE) reflects the fidelity of the reconstructed isopotential maps at each time point. The effects of cardiac rigid motion on the reconstructed EGMs morphology were evaluated using maximum values, minimum values, and peak times, as illustrated in Figure 1D. The maximum and minimum values refer to the peak voltage values within the entire QRST data cycle. The peak time (PT) corresponds to the moment when the maximum peak voltage of the QRS complex occurs.

## 3 Results

### 3.1 Translation vs. inverse solution

**FIGURE 2:**
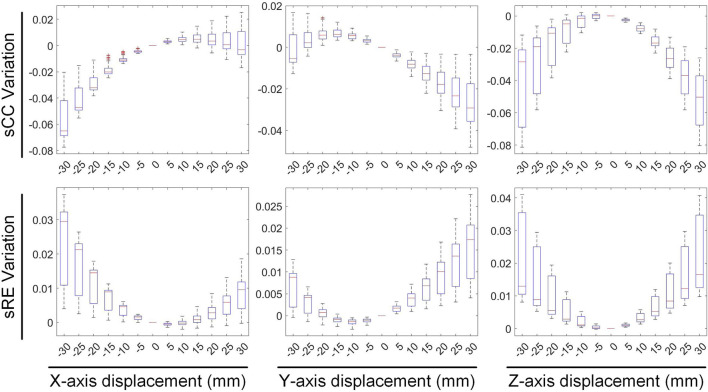
The influence of translation on the sCC and sRE of the inverse solution in ECGI.

**FIGURE 3:**
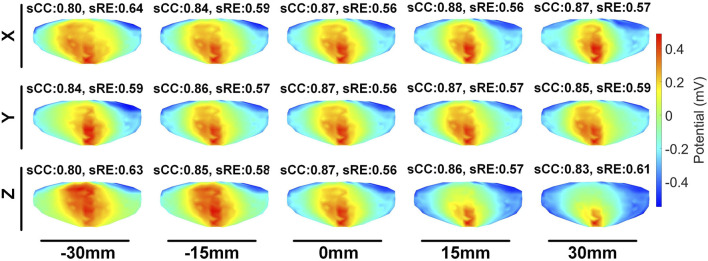
An examples of ECGI inverse reconstruction with different translation distances (time instant = 8). Estimated values of the pericardial cage potential when the cage is translated along the X, Y, and Z axes by −30 mm, −15 mm, 15 mm, and 30 mm, respectively.

We first evaluated the impact of translating a heart model along coordinate axes on the accuracy of ECGI inverse solutions. Figure 2 presents the variations in inverse-solved sCC and sRE following displacements along the three axes within the range of −30 mm to 30 mm. Overall, as the displacement increased, the sCC decreased while the sRE increased; for displacement deviations within 30 mm on the target dataset, the changes in sCC are less than 8%, and those in sRE were less than 4%. In Figure 2, compared to the scenario with no displacement, when moving −30 mm along the X, Y, and Z axes respectively, the sCC decreased by 4.89% ± 2.85%, −0.20% ± 1.47%, and 4.66% ± 3.47%, while the sRE increased by 2.07% ± 1.67%, 0.62% ± 0.66%, and 2.46% ± 1.65%. For displacements of 30mm, the sCC decreased by −0.42% ± 2.11%, 2.57% ± 2.23%, and 5.31% ± 2.72%, while the sRE increased by 0.92% ± 0.94%, 1.59% ± 1.18%, and 2.52% ± 1.55%. Notably, when moving along the positive X-axis and the negative Y-axis, the sCC initially increased slightly before decreasing. In contrast, displacements along the Y-axis have the minimal impact on the inverse solution. Figure 3 presents an example of ECGI spatial inverse reconstruction at various X, Y, and Z displacement distances, with a time instant equal to 8.

**FIGURE 4:**
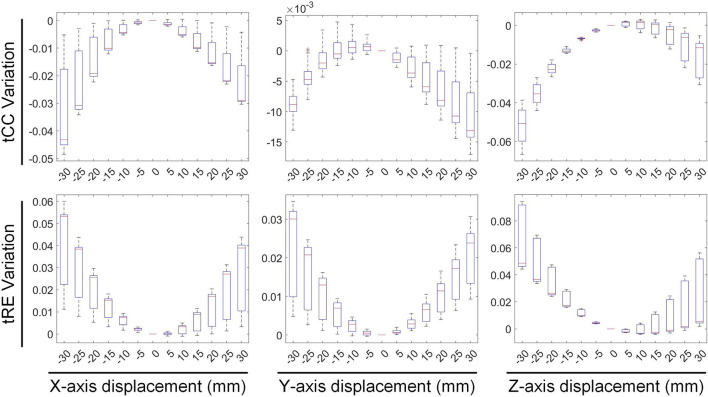
The influence of translation on the tCC and tRE of the inverse solution in ECGI.

**FIGURE 5:**
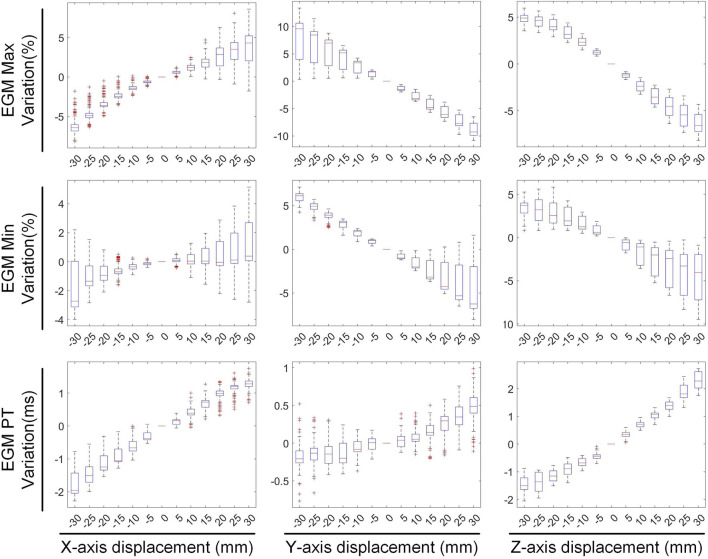
The effect of translation on the maximum, minimum and PT of EGM.

Figures 4, 5 illustrate the impact of translation on the temporal dimension of the inverse solution in ECGI. Figure 4 reveals that, in the case of a −30 mm translation, the inverse-solved tCC decreases by 2.68% ± 2.16%, 0.89% ± 0.42%, and 5.26% ± 1.4% along the X, Y, and Z axes, respectively, while the tRE increases by 3.56% ± 2.44%, 1.97% ± 1.49%, and 6.94% ± 2.51%, respectively. Similarly, for a 30 mm translation, the tCC decreases by 1.73% ± 1.30%, 0.87% ± 0.83%, and 1.80% ± 1.26% along the X, Y, and Z axes, respectively, while the tRE increases by 2.35% ± 2.03%, 2.00% ± 1.07%, and 2.91% ± 2.72%, respectively. Notably, unlike the sCC, there is a slight increase in tCC when moving along the positive Z-axis, which is an interesting observation that warrants further investigation.

Figure 5 illustrates the variations in the maximum value, minimum value, and PT of the inverse-solved EGM under different displacements. When translated by −30 mm along the X, Y, and Z axes, the maximum values of the EGM changed by −4.98% ± 3.19%, 6.84% ± 6.51%, and 4.75% ± 1.21%, respectively; the minimum values changed by −0.90% ± 3.09%, 5.69% ± 1.44%, and 3.05% ± 2.22%, respectively; and the PTs changed by −1.53 ± 0.75 ms, −0.12 ± 0.64 ms, and −1.47 ± 0.59 ms, respectively. Similarly, under a 30 mm translation, the maximum values of the EGM varied by 3.40% ± 5.18%, −8.67% ± 2.16%, and −6.24% ± 1.92%, respectively; the minimum values changed by 1.17% ± 3.97%, −3.21% ± 4.82%, and −5.17% ± 4.30%, respectively; and the PTs shifted by 1.23 ± 0.51 ms, 0.44 ± 0.55 ms, and 2.24 ± 0.48 ms, respectively. Moving along the positive direction of the X-axis increases the maximum value of the inverse solution of EGM and shifts the PT later. Moving along the Y and Z-axis directions decreases both the maximum and minimum values of the inverse solution of EGM, with a slight shift in PT to later. Overall, when the heart position moves within a range of −30 mm to 30 mm, the changes in the maximum and minimum values of the inverse-solved EGM are less than 10%, and the changes in PT generally are less than 2 ms.

### 3.2 Rotation vs. inverse solution

**FIGURE 6:**
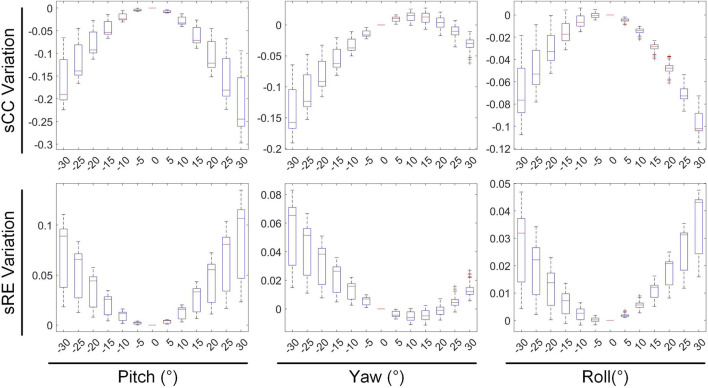
The influence of rotations on the sCC and sRE of the inverse solution in ECGI.

**FIGURE 7:**
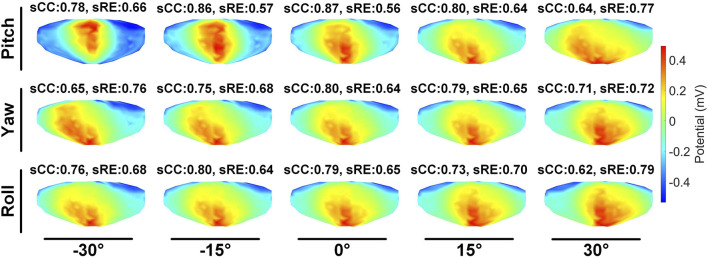
An example of ECGI inverse reconstruction with different rotation angles (time instant = 8).

The second stage involves assessing the impact of heart model rotations on the accuracy of the inverse solution in ECGI. Figure 6 illustrates the changes in sCC and sRE of the inverse solution after rotation between −30° and 30° around the each of the three axes. Similar to translation, the quality of the inverse solution generally exhibits a downward trend as the rotation angle increases. In Figure 6, compared to the conditions with 0° of pitch, yaw, and roll rotations, under a −30° rotation, the sCC decreased by 14.48% ± 7.93%, 12.78% ± 6.32%, and 6.30% ± 4.46%, respectively, while the sRE increased by 6.44% ± 4.61%, 4.90% ± 3.40%, and 2.56% ± 2.13%, respectively. In the case of a 30° rotation, the sCC decreased by 19.57% ± 10.14%, 3.63% ± 2.55%, and 9.39% ± 2.11%, respectively, and the sRE increased by 7.90% ± 5.59%, 1.62% ± 1.05%, and 3.17% ± 1.58%, respectively. Notably, as the yaw angle increases positively, the sCC first increases slightly before decreasing, with the sRE following a synchronous pattern of first decreasing and then increasing. Figure 7 demonstrates an example of the inverse reconstruction in the ECGI space under different rotational angles for pitch, yaw, and roll, at a specific time instant equal to 8.

**FIGURE 8:**
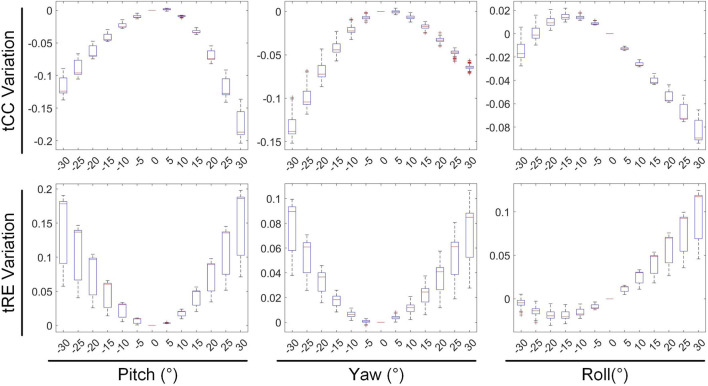
The influence of rotations on the tCC and tRE of the inverse solution in ECGI.

**FIGURE 9:**
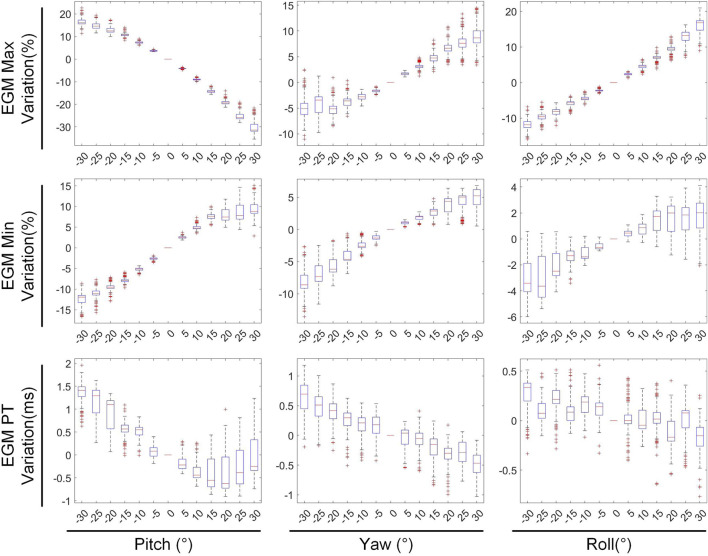
The effect of rotations on the maximum, minimum and PT of EGM.

In Figures 8, 9, the impact of rotation on the temporal dimension of ECGI inverse solutions is described. In Figure 8, compared to the case without rotation, under rotations of −30° for pitch, yaw, and roll, the tCC decreased by 11.34% ± 2.40%, 12.52% ± 2.65%, and 1.10% ± 1.66%, respectively, while the tRE increased by 12.40% ± 6.66%, 6.87% ± 3.08%, and −0.67% ± 1.20%, respectively. Under rotations of 30°, the tCC decreased by 17.00% ± 3.37%, 6.40% ± 0.78%, and 7.95% ± 1.42%, respectively, while the tRE increased by 13.45% ± 6.33%, 6.71% ± 3.95%, and 8.52% ± 3.93%, respectively. Additionally, as the roll angle varies from 0° to −30°, the tCC exhibits a slight initial increase followed by a decrease, with an average magnitude of change less than 3%.

Figure 9 depicts the changes in the maximum value, minimum value, and PT of the inverse solution of EGM under different rotation angles. When the pitch, yaw, and roll rotation angles are −30°, the maximum values of EGM change by 17.01% ± 5.64%, −4.30% ± 6.70%, and 11.40% ± 4.51% respectively, the minimum values change by −12.67% ± 4.01%, −8.14% ± 5.43%, and −2.70% ± 3.28% respectively, and the PTs shift by 1.29 ± 0.67 ms, 0.49 ± 0.69 ms, and 0.09 ± 0.42 ms respectively. When the rotation angles are 30°, the maximum values of EGM change by −28.54% ± 6.86%, 8.91% ± 5.54%, and 14.99% ± 5.99% respectively, the minimum values change by 8.97% ± 6.09%, 3.67% ± 3.15%, and 1.02% ± 3.09% respectively, and the PTs shift by 0.25 ± 0.99 ms, −055 ± 0.47 ms, and −0.26 ± 0.51 ms respectively. During positive pitch rotation, the maximum value of the EGM decreases, while the minimum value increases. When yaw and roll rotate in the positive direction, the extreme values (both maximum and minimum) increase. Additionally, as the pitch and yaw rotation angles intensify, the average PT advances forwards. Conversely, the PT remains relatively stable when subjected to roll rotations. Overall, when the heart’s rotation angles move within a range of −30° to 30°, the changes in the maximum and minimum values of the inverse solution of EGM are typically less than 20%, and the changes in PT are generally within 2 ms.

### 3.3 Summary of statistical tests

In this section, we summarize the statistical results presented in Sections 3.1 and 3.2, as shown in Table 1. Given a 30 mm displacement and 30° rotation of heart, greater emphasis should be placed on the impact of heart rotation, as the alterations in CC, RE, PTs and the peak values of EGMs are more pronounced compared to those caused by translational motion. During translational motion along the coordinate axes, movement along the Y-axis (anteroposterior motion within the chest cavity) exhibits an overall smaller influence on CC and RE compared to movement along the XZ axes. In terms of rotational motion, roll has the least impact on CC and RE, followed by yaw, with pitch having the greatest impact. Upon moving within a range of −30 mm to 30 mm, the changes in the peak values of the EGMs are generally less than 10%. Similarly, upon rotating within a range of −30° to 30°, the changes in the peak values of EGMs are typically less than 20%. Notably, When moving or rotating within a range of 30 mm or 30°, the change in PT generally does not exceed 2 ms.

## 4 Discussion

In this study we employed a dataset from the University of Utah in order to quantify how cardiac positional movements affect the reconstruction of ECGI. Utilizing the zero-order Tikhonov regularization method, we conducted ECGI reconstructions and evaluated the impact of six different cardiac motion patterns, including translations and rotations, designed based on clinical experience. Experimental results indicated that among displacements of ±30 mm and rotations of ±30°, rotations have a greater influence on ECGI reconstruction. For the same displacement distance, movement along the Y-axis exert the least impact on ECGI reconstruction. Similarly, among rotations of the same angle, rolling has a smaller effect on the reconstruction. Furthermore, we found that changes in the CC value are typically less than 2% for a 10 mm displacement and less than 5% for a 10° rotation, suggesting that within a certain range, the variations in ECGI due to cardiac geometric motion can be considered neglected.

### 4.1 Accuracy of the inverse problem solution

The correlation between the inverse solution of ECGI and intracardiac contact EGMs, whether experimentally or simulationally measured, is poor (Figuera et al., 2016). When considering the absence of any displacement, the inverse solution derived from our dataset yields the following results: sCC = 0.60 ± 0.11, sRE = 0.84 ± 0.07, tCC = 0.85 ± 0.02, and tRE = 0.68 ± 0.06. It is evident that the temporal correlation of epicardial potential reconstruction is stronger than the spatial correlation, with the epicardial EGM exhibiting relatively superior fidelity in terms of morphology. Errors in epicardial potential reconstruction stem from a multitude of factors, including the positioning and quantity of body surface electrodes, the precision of the heart model, the inherently ill-conditioned nature of the inverse problem, and external interferences (Bergquist et al., 2021a). We employed the Tikhonov zero-order regularization method for the inverse calculation. While this method utilizes an L2-norm-based penalty function, which tends to produce a smooth EGM, it is the most widely used and straightforward to implement (Figuera et al., 2016). However, the sensitivity of alternative regularization methods to rigid displacements necessitates further exploration.

Moving beyond regularization methods, the ECGI inverse problem necessitates the acquisition of MRI or CT images to establish an accurate patient-specific anatomical model. These MRI or CT images are captured with BSP electrodes in place, ensuring the preservation of geometric parameters (Rodrigo et al., 2018). However, it is not always feasible to obtain dynamic MRI together with useful BSP recordings. Our study reveals the performance of ECGI technology under varying cardiac motion conditions by quantifying the impact of cardiac translation and rotation on the accuracy of ECGI inverse solutions. Experimental data indicate that, although cardiac motion does exert a certain influence on inverse solution results, within a specific range (such as 10 mm of displacement and 10° of rotation), this influence is relatively insignificant, with typical changes in CC less than 2% and 5%, respectively. This underscores the high stability of ECGI inverse solutions under certain conditions.

In the study by Rodrigo et al., which aimed to correct inaccuracies in anatomical models by maximizing reconstruction quality, it was observed that the average positioning error of the atrium on the XYZ axes within the mathematical model was 1.7 ± 2.4 mm. In contrast, the average error in patient data was 9.1 ± 11.5 mm (Rodrigo et al., 2017; Rodrigo et al., 2018). Gisbert et al. employed an automatic optimization algorithm to localize atrial anatomical structures, achieving a positional deviation of 0.5 ± 0.5 cm (Gisbert et al., 2020).These findings concurs with our results, indicating that the inverse problem can accommodate spatial uncertainties up to 1 cm. Furthermore, Coll-Font and Brooks’ investigation into tracking heart position using body surface potentials (Coll-Font and Brooks, 2018), alongside Bergquist et al.'s research on heart position uncertainty (Bergquist et al., 2022; Bergquist et al., 2023), reinforces the notion that appropriate spatial positioning and the construction of precise anatomical models enhance the outcomes of the inverse solution. Consequently, slight variations in heart position do not appreciably impact clinical decision-making when the errors stemming from heart position uncertainty are smaller than other errors inherent in the ECGI system.

### 4.2 Impact of cardiac motion on the inverse solution

Although our findings indicate that cardiac motion within a certain range has a limited impact on the accuracy of the ECGI inverse solution, understanding how these motions specifically affect the inverse solution process remains crucial. We found that movements along the Y-axis in the anterior-posterior direction of the thorax have a relatively minor impact on the ECGI inverse solution. This may be attributed to the dense arrangement of electrodes in the anterior-posterior plane of the thorax, which are in closer proximity to the heart. Consequently, movements along the Y-axis exert less influence on the relative positioning between the body surface electrodes and the heart, thereby reducing the reconstruction error in potential distribution. In the study by Rodrigo et al., the positioning errors of the atrium on the XYZ axes within the mathematical model, were found to be 1.0 ± 2.1 mm, 3.0 ± 2.6 mm, and 1.0 ± 2.1 mm, respectively (Rodrigo et al., 2018). Our research aligns with the findings of Rodrigo et al., indicating that the ECGI system is least sensitive to errors along the Y-axis, resulting in the poorest localization performance on the Y-axis. Additionally, we observed consistency in the maximum and minimum values of the inverse solution EGM when moving in the positive direction along the coordinate axes, with these values changing almost synchronously. As the heart position shifts towards the upper left side within the chest cavity (i.e., the positive direction of the coordinate axes), the PTs of the inverse solution EGM undergo a gradual delay.

Compared to translational motion, rotational motion has a more significant impact on the ECGI inverse solution. This may be because rotations of the heart model cause more drastic changes in the relative position between the heart and body surface electrodes compared to translations. Among ±30° rotational movements, rolling exhibits the least impact on the inverse solution, with the impact on CC typically being less than half of that observed for yaw and pitch. This may be due to the synchronous movement of the heart model relative to the torso model during roll, resulting in a more regular pattern of model coordinate transformation compared to yaw and pitch. Notably, during pitch motion, as the rotation angle increases in the positive direction, the maximum value of the inverse-solved ECG decreases, the minimum value increases, and the PTs shifts forwards. In contrast, as the rotation angle increases in the positive direction for roll and yaw motions, the extreme values of the inverse-solved EGM increase, while the PTs remain almost unchanged. These differences may be attributed to the yaw motion model having a 15° pitch angle and the roll motion being performed under conditions of pitch = 15° and yaw = 15°.

It is noteworthy that almost all models are not in the optimal position for CC in the absence of displacement. When the heart geometry model is translated along the X, Y, and Z axes within the range of −30 mm to 30 mm, the average improvement in the CC of the inverse solution does not exceed 0.8%, with a maximum value not exceeding 2.5%. Through a linear search within the three-dimensional XYZ space, with a step size of 1 mm, we found that the average increase in the CC of the inverse solution is 1.36%, and the maximum increase is 2.95%. This further demonstrates the robustness of the ECGI inverse problem to variations in heart position, as slight perturbations in heart position do not significantly affect the accuracy of the ECGI inverse solution.

### 4.3 Regional inverse solution discrepancies

**FIGURE 10:**
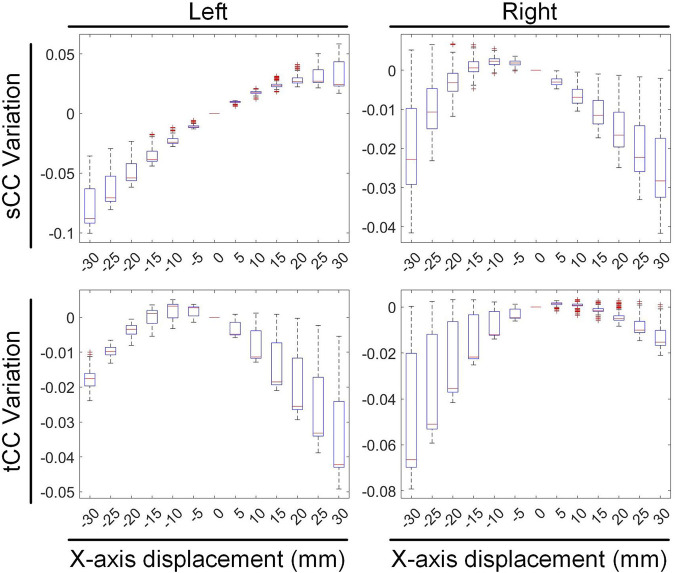
The influence of X-axis translational motion on the inverse solutions of the left and right cardiac regions.

We conducted a preliminary investigation on the sensitivity of inverse solutions in different heart anatomical regions to various motion types. Specifically, during translational motion along the X-axis, we segmented the heart structure into left and right halves along the midline and independently quantified the changes in sCC and tCC within these two segments, as illustrated in Figure 10. Our findings revealed that upon leftward displacement of the heart (positive X-axis movement), the sCC in the left segment initially increases and then decreases, while the tCC shows a steady decline. In the right segment, the sCC decreases continuously, and the tCC exhibits a slight increase followed by a gradual decline. When the heart moves rightward (negative X-axis movement), the sCC in the left segment decreases steadily, while the tCC first rises and then falls. In the right segment, the sCC shows an initial increase followed by a decrease, and the tCC decreases steadily. Collectively, the sCC and tCC in the left and right segments exhibit nearly symmetrical, mirror-like variations.

In Figure 10, compared with the no-displacement condition, under a −15 mm X-axis displacement (rightward movement), the sCC and tCC values in the left segment changed by −3.08% ± 1.33% and −0.09% ± 0.45%, respectively. Conversely, in the right segment, the sCC and tCC values changed by 0.09% ± 0.56% and −1.11% ± 1.42%, respectively. Under a 15 mm X-axis displacement (leftward movement), the sCC and tCC in the left segment changed by 2.46% ± 0.66% and −1.00% ± 1.09%, respectively, while in the right segment, they changed by −0.91% ± 0.82% and −0.16% ± 0.43%, respectively. It is worth noting that with the displacement of the heart, an increase in sCC is usually accompanied by a decrease in tCC. To illustrate, upon a slight leftward shift of the heart, the sCC in the left segment increases while the tCC decreases; conversely, in the right segment, the sCC decreases as the tCC increases. A similar phenomenon is observed in the right segment during rightward movement.

**FIGURE 11:**
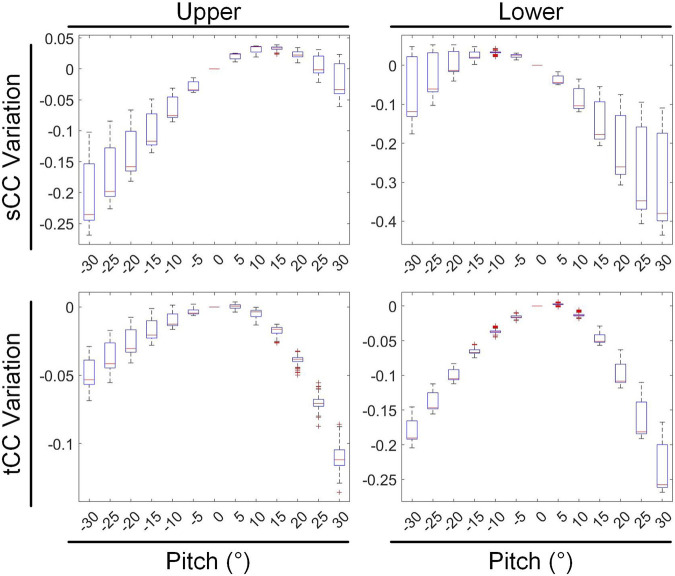
The influence of pitch rotational motion on the inverse solutions of the top and bottom cardiac regions.

During pitch rotational movement, we have divided the cardiac structure into two distinct sections: the upper segment, which corresponds to the base of the heart, and the lower segment, which corresponds to the apex. Figure 11 illustrates the variations in sCC and tCC within the upper and lower segments of the cardiac structure as the heart undergoes pitch rotational movement from −30° to 30°. In Figure 11, compared to 0° rotation, at a pitch rotation of −15°, the sCC and tCC values in the upper segment changed by −9.22% ± 4.35% and −1.46% ± 1.35%, respectively, while those in the lower segment changed by 2.50% ± 2.26% and −6.48% ± 0.98%. In contrast, at a pitch rotation of 15°, the sCC and tCC values in the upper segment altered by 3.12% ± 0.77% and −1.95% ± 0.69%, respectively, while those in the lower segment changed by −13.04% ± 7.57% and −4.28% ± 1.39%. A notable observation from Figure 11 is that as the pitch rotation angle of the heart increases positively, the sCC in the upper segment initially increases and then decreases, whereas the sCC in the lower segment exhibits a monotonic decrease. In contrast, when the pitch rotation angle increases negatively, the sCC in the lower segment first increases and then decreases, while the sCC in the upper segment shows a monotonic decrease, creating nearly mirror image trends in both cases. Throughout the entire pitch rotation range, the tCC trends in both the upper and lower segments are similar, with both decreasing as the pitch angle increases. However, it is crucial to highlight that in most instances, the influence of pitch rotational movement on the lower segment of the heart is considerably more pronounced than on the upper segment, with the range of CC changes in the lower segment being nearly double that observed in the upper segment.

### 4.4 Limitations and future work

Although our study provides a comprehensive evaluation of the impact of cardiac translational and rotational motions on the accuracy of ECGI inverse solution, it is not devoid of inherent limitations. In real-world scenarios, cardiac motion is considerably more complex. Factors such as the non-rigid deformation of the heart, respiratory motion, and cardiac contraction may exert more intricate and unpredictable influences on the ECGI inverse solution (Jiang et al., 2008). Specifically, during the heartbeat movement that encompasses the entire duration of systole, the heart experiences ventricular shortening along its long axis, accompanied by corresponding torque variations around this axis. Consequently, the ventricular base and apex exhibit opposing movements (Pullan et al., 2010). This dynamic process not only shifts the heart’s position but also modifies its dimensions and contour. Additionally, during the process of respiration or alterations in body posture, the heart undergoes a rotational movement around its anchor point, induced by the movements of the ribs and/or diaphragm. Typically, this rotational movement persists for a duration that extends beyond a single heartbeat cycle (Koley, 2024). These more complex motion patterns, characterized by their dynamic and nonlinear nature, pose significant challenges to the advancement of ECGI technology and merit further in-depth investigation (Jiang et al., 2009).

Furthermore, the dataset for this study was derived from the Langendorff perfusion system, which simplifies the simulation of torso conductor volume by excluding structures such as the lungs (Bergquist et al., 2021b). This simplification may introduce deviations between the model and the actual human body. The interaction between the inhomogeneity of the thoracic volume and the uncertainty in heart position has not been thoroughly explored in this study. However, according to relevant literature, although inhomogeneity of the thoracic volume may exert some influence on the reconstruction of epicardial potentials, electrograms, and activation sequences, its impact is relatively minor and generally negligible in clinical practice (Bear et al., 2015; Rudy, 2015). This conclusion provides some support for the rationality of our study, but further validation and refinement are still required in the future.

It is noteworthy that the same forward solver was employed for generating BSP signals and performing inverse calculations in this study. Although this approach simplifies the research process, it may also lead to overconfidence in the inverse solution results. To mitigate this potential risk, white noise was deliberately added to the generated BSP signals to simulate noise interference in real environments. This practice aligns with standard practices in research within this field (Rodrigo et al., 2018; Gisbert et al., 2020) and contributes to enhancing the reliability and applicability of the research findings. Despite the inevitable limitations introduced by the use of synthetic BSP data, the dataset provided by this study allows for precise control over heart motion, which is often difficult to achieve in experimental settings, thereby limiting the depth and breadth of experimental research. To a certain extent, synthetic data compensates for the deficiencies of experimental research.

Lastly, the variations in the inverse solution of ECGI due to cardiac motion investigated in this study were only tested under sinus rhythm, and their extension to arrhythmic conditions remains unverified. Arrhythmias are diverse in types and electrical patterns, and their impacts on ECGI inverse solution changes caused by heart displacement may vary. While we hope that the general conclusions of this study apply to a broader range of data, including both sinus rhythm and various arrhythmic conditions, their reliability under other conditions still awaits testing.

## 5 Conclusion

Heart motion is closely related to the performance of ECGI reconstruction. Our research findings reveal that, among the translational and rotational motions of the heart, rotational motion should be prioritized due to its significantly stronger impact on the changes in CC and RE of the ECGI inverse solution compared to translational motion. Among the translational motions along the coordinate axes, movement along the y-axis (anteroposterior motion within the chest cavity) has the least impact. In terms of rotational motions, roll has the smallest impact, yaw follows, and pitch has the largest. The ECGI inverse problem exhibits a certain degree of robustness to heart position, with CC changes being less than 2% for a 10 mm displacement and less than 5% for a 10° rotation. This implies that within a certain range, the ECGI changes resulting from geometric heart motion can be disregarded, and it is not necessary to pursue extremely high precision in patient-specific thoracic-heart geometry for practical applications.

## Data Availability

Publicly available datasets were analyzed in this study. This data can be found here: https://www.ecg-imaging.org/edgar-database. Upon reasonable request, the raw data supporting the conclusion of this article will be made available by the authors, without undue reservation.
